# Associations between fatty infiltration of rotator cuff muscles and tear size or location of rotator cuff tendon

**DOI:** 10.3389/fsurg.2024.1416921

**Published:** 2024-08-22

**Authors:** Shiro Kajiyama, Ko Chiba, Tatsunari Aoki, Kiyoshi Sada, Shuntaro Sato, Makoto Osaki

**Affiliations:** ^1^ Department of Sports Medicine Center, Nagasaki University Hospital, Nagasaki, Japan; ^2^ Department of Orthopaedic Surgery, Graduate School of Biomedical Sciences, Nagasaki University, Nagasaki, Japan; ^3^ Department of Orthopaedic Surgery, Jikei Hospital, Isahaya, Japan; ^4^ Clinical Research Center, Nagasaki University Hospital, Nagasaki, Japan

**Keywords:** rotator cuff tear, fatty infiltration, Goutallier classification, Lafosse classification, Cofield classification, tear size, tear location

## Abstract

**Background:**

Fatty infiltration (FI) of rotator cuff muscles in patients with rotator cuff tears is an important imaging factor for determining surgical indications. However, the associations between FI grade and the size or location of adjacent rotator cuff tears are not well-known. This study aimed to primarily determine whether tear size and location, especially for the SSc tendon, are associated with FI of adjacent rotator cuff muscles. The secondary aim was to clarify which patient factors are associated with rotator cuff muscle FI in rotator cuff tear cases.

**Methods:**

This study examined 373 shoulders of 348 patients (264 males and 109 females; mean age of 62.8 years) who underwent arthroscopic rotator cuff surgery. The FI grades of the supraspinatus (SSP), infraspinatus (ISP), and subscapularis (SSc) muscles were assessed using preoperative magnetic resonance imaging (MRI) using the Goutallier classification modified by Fuchs. According to the preoperative MRI and intraoperative findings, the tear size of the posterior–superior rotator cuff (SSP–ISP) was classified using a modified six-grade scale of the Cofield classification, and that of the SSc tear was classified using a six-grade scale according to the Lafosse classification. Age at surgery, sex, body mass index (BMI), presence of diabetes mellitus (DM) or hyperlipidemia (HL), trauma history, and duration of symptoms were investigated.

**Results:**

The FI grades of the SSP, ISP, and SSc were significantly associated with the size of the tears in those muscles (all *P *< 0.01). Furthermore, the FI grades of the SSP and the ISP were significantly associated with SSc tear size (*P *< 0.01), and the FI grade of the SSc was significantly associated with SSP–ISP tear size (*P *< 0.01). Patient age at surgery was significantly associated with FI grade (*P *< 0.01), with significant progression of the FI grade with advancing age. However, there were no significant associations between the FI grade and sex, BMI, presence of DM or HL, trauma history, and duration of symptoms.

**Conclusions:**

The FI grade of each of the rotator cuff muscles is affected by not only the tear severity of the muscle concerned but also by the severity of any tear in the adjacent rotator cuff.

## Background

The use of preoperative magnetic resonance imaging (MRI) to assess fatty infiltration (FI) and muscle atrophy of the rotator cuff muscles is important in surgical planning for rotator cuff tears, and the most widely used FI assessment method is the Goutallier classification modified by Fuchs using MRI (1–4). Progression is thought to be affected by several different factors, of which the relationship with rotator cuff tear size in particular has been widely reported (5–7). Supraspinatus (SSP) tendon injury is the most common form of rotator cuff tear, and it is thought that, as the tear widens, the damage extends to the infraspinatus (ISP) tendon and the subscapularis (SSc) tendon. FI of the rotator cuff muscles has been shown to progress as the tear enlarges (8).

**Figure 1:**
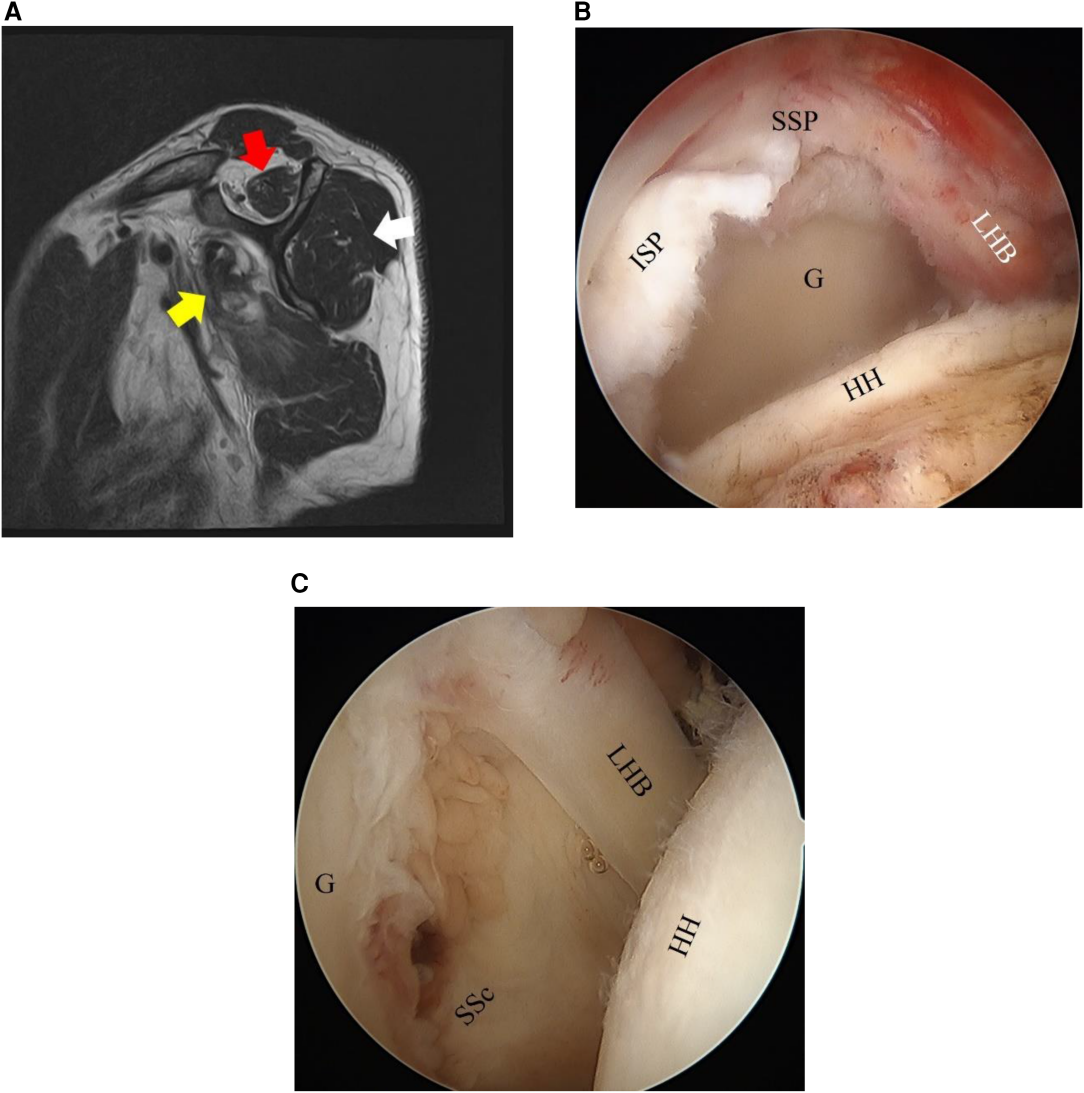
Fatty infiltration (FI) of the rotator cuff muscles on MRI sagittal images and arthroscopic findings of Case 1. **(A)** A 64-year-old man. Right rotator cuff tear. The FI grades of the SSP (red arrow), ISP (white arrow), and SSc (yellow arrow) are 2, 1, and 2, respectively. **(B,C)** Based on the arthroscopic findings, the SSP–ISP tear is graded as a massive tear and the SSc tear as Lafosse Type IV. SSP, supraspinatus tendon; ISP, infraspinatus tendon; SSc, subscapularis tendon; G, glenoid; LHB, long head of biceps; HH, humeral head.

**Figure 2:**
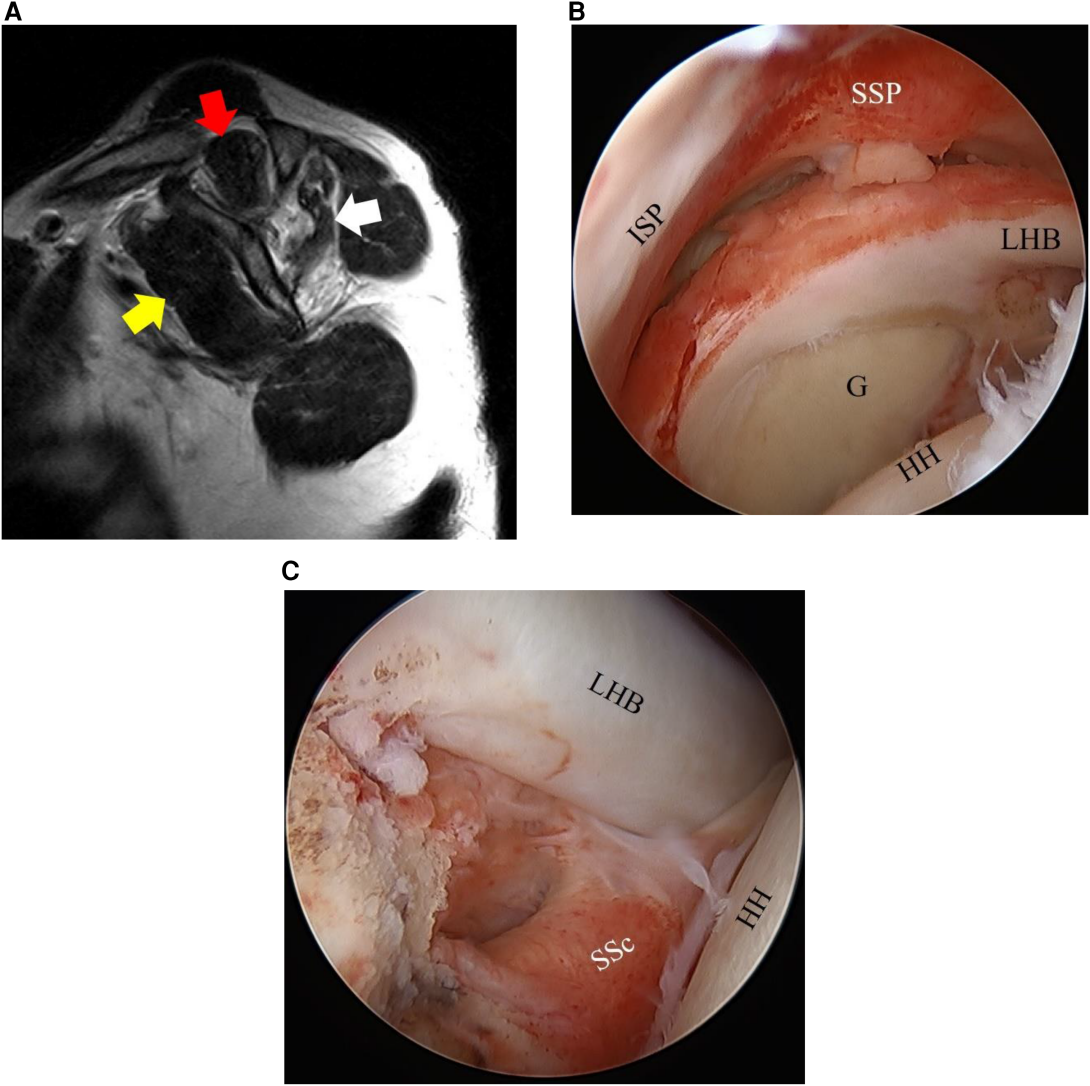
Fatty infiltration (FI) and arthroscopic findings of Case 2. **(A)** A 43-year-old man. The FI grades of the SSP (red arrow), ISP (white arrow), and SSc (yellow arrow) are 2, 3, and 0, respectively. **(B,C)** Based on the arthroscopic findings, the SSP–ISP tear is graded as a massive tear and the SSc tear as Lafosse Type I. SSP, supraspinatus tendon; ISP, infraspinatus tendon; SSc, subscapularis tendon; G, glenoid; LHB, long head of biceps tendon; HH, humeral head.

In recent years, the attachments of the rotator cuff muscles have been the subject of in-depth anatomical studies (9, 10) that have identified the anatomical importance of the anterior–superior part. It is expected that disruption of the anterior–superior rotator cuff attachment affects muscle FI in both SSc and SSP over time. In clinical settings, there have been cases in which FI of the SSP and ISP muscles was more advanced than expected or cases in which it was better than expected, when compared with the status of the torn tendon based on the medical history and preoperative MRI (Figures 1A, 2A). The relationship between the FI of each muscle and anterior–superior tear size, especially in SSc tendon tears, has not yet been clarified. We hypothesized that in such cases, the progression of the SSc tear affects muscle FI of the adjacent rotator cuff.

This study primarily aimed to determine whether tear size and location, especially in the SSc tendon, are associated with FI of adjacent rotator cuff muscles. The secondary aim was to clarify which patient factors, such as patient age, comorbidities, or trauma history, are associated with rotator cuff muscle FI.

## Materials and methods

### Study participants

The study examined 373 shoulders of 348 patients (mean age at surgery, 62.8 years; range, 31–80 years). Patients who underwent arthroscopic surgery (primary repair, fascia lata autograft, partial repair, or debridement only) for rotator cuff tears at our hospital between April 2013 and August 2022, in whom FI could be assessed on preoperative MRI in the oblique sagittal image, were included. The exclusion criteria were cases of reoperation, cases of paralysis due to other diseases, cases of rotator cuff tear associated with shoulder dislocation, and cases of rotator cuff tear-related arthropathy for which reverse shoulder arthroplasty was indicated. There were 264 male shoulders and 109 female shoulders. The following patient factors were investigated for each participant: age at surgery, sex, body mass index (BMI), presence of diabetes mellitus (DM) or hyperlipidemia (HL), trauma history, and time from the appearance of symptoms to surgery (duration of symptoms). The time from trauma to MRI (in traumatic cases) was also investigated.

### Evaluation of fatty infiltration (FI) and tear size

The FI grades of the SSP, ISP, and SSc were assessed on preoperative MRI according to the Goutallier classification modified by Fuchs (1–3). The assessment was conducted using the most lateral sagittal slice in which the scapular spine was continuous with the scapular body, the so-called Y-shaped view (1, 3) (Figures 1A, 2A). The field of view was 16 × 16 cm, and the thickness was 5 mm, for the sagittal sections. Grading of the fatty infiltration was as follows: Stage 0, no fatty infiltration; Stage 1, some fatty steaks; Stage 2, less fat than muscle; Stage 3, as much fat as muscle; and Stage 4, more fat than muscle (1, 3). Cysts around the rotator cuff muscle in continuity with the rotator cuff tear or glenohumeral joint were excluded from the assessment. All assessments were conducted by a single shoulder surgeon (lead author). To test reliability, 10 of the patients were randomly selected, and the Goutallier classifications of the SSP, ISP, and SSc muscles of these 10 patients were independently assessed twice by two experienced shoulder surgeons.

Surgeries were performed by four shoulder surgeons, with one senior surgeon (lead author) supervising the surgeries of the three other surgeons. Surgery was conducted under general anesthesia with the patient in the beach chair position. The size of the posterior–superior (SSP–ISP) tear was classified on a six-grade scale according to the modified Cofield classification (11) based on preoperative MRI evaluation and intraoperative arthroscopic findings. If only the SSc was torn, this was classified as no tear, and the tears were classified as a partial tear (bursal side or joint side), small tear (<1 cm), medium tear (<1–3 cm), large tear (3–5 cm), or massive tear (>5 cm). The SSc tear was classified on a six-grade scale according to the Lafosse classification (12) with arthroscopic findings, with the humerus in slight flexion and neutral rotation in the beach chair position, and preoperative imaging analysis (Type 0, no tear; Type I, partial superior third lesion; Type II, complete superior third lesion; Type III, complete superior two-thirds lesion; Type IV, complete tendon lesion with well-centered humeral head and FI graded ≤3; Type V, complete tendon lesion with subluxated humeral head, coracoid conflict, and FI graded ≥3) (Figures 1B,C, 2B,C).

### Statistical analysis

Cohen's *κ* statistics were calculated for both intraobserver and interobserver reliabilities of the Goutallier classification. To investigate the associations between the FI grades of each rotator cuff muscle and SSP–ISP and SSc tear size, we performed a linear-by-linear association test (13) to assess whether SSP–ISP and SSc tear severities tended to be higher when the FI grade for each rotator cuff muscle was higher. The associations between FI grade and sex, the presence of DM or HL, and trauma were investigated by the Mann–Whitney *U*-test, and the associations between FI grade and age, BMI, duration of symptoms, and trauma–MRI duration were investigated by Spearman's rank correlation coefficient. Age, BMI, period since symptom onset to surgery, and trauma–MRI duration were stratified by 10-year, 5 kg/m^2^, 6-month, and 3–6-month intervals, respectively, and the relationships with muscle FI were examined. Two-sided *P*-values are reported. Significance was determined at a *P*-value of <0.05. Statistical analyses were performed using Statcel 3 (OMS Publications, Tokyo, Japan) and R4.2.1 (The R Foundation for Statistical Computing, Vienna, Austria).

## Results

The mean age of the study participants was 62.8 years, with 46.6% in the 60–69-year age group, and 70.8% were men. The mean BMI was 24.0 kg/m^2^, and 68% of participants had a BMI ≤25.0 kg/m^2^. Approximately 12% of cases had DM and HL (statins were administered at the time of surgery in 70% of HL cases). A trauma history was reported in 39.4% of the participants. The mean duration of symptoms was 7.9 months, and this duration was ≤6 months in 63.8% of participants (Table 1). In terms of SSP–ISP tear size, the medium tear group was the largest, with 158 shoulders (42.4%), followed by the large tear group, with 105 (28.1%). SSc tear size according to the Lafosse classification was “no tear” in 119 shoulders (31.9%), and the largest group among those shoulders for which a tear was observed was Type II, in 101 shoulders (27.1%) (Table 1).

**Table 1:** Patients’ characteristics.

Age, year and Mean (SD); 62.8 (8.7)	30–39	1.6% (6)
	40–49	5.9% (22)
	50–59	22.3% (83)
	60–69	46.6% (174)
	70–79	23.1% (86)
	80–	0.5% (2)
Sex	Male	70.8% (264)
	Female	29.2% (109)
BMI, kg/m^2^ and Mean (SD); 24.0 (3.3)	−20.0	9.7% (36)
	20.1–25.0	58.2% (217)
	25.1–30.0	28.4% (106)
	30.1–	3.7% (13)
DM	DM	12.3% (46)
	Non-DM	87.7% (327)
HL	HL	12.9% (48)
	Non-HL	87.1% (325)
Trauma	Traumatic	39.4% (147)
	Non-traumatic	60.6% (226)
Duration of symptoms, months and Mean (SD); 7.9 (10.0)	−6	63.8% (238)
	7–12	24.9% (93)
	13–24	7.8% (29)
	25–	3.5% (13)
SSP–ISP tear size and (Modified Cofield classification)	No tear	1.3% (5)
	Partial	18.0% (67)
	Small	2.7% (10)
	Medium	42.4% (158)
	Large	28.1% (105)
	Massive	7.5% (28)
SSc tear severity and (Lafosse classification, type)	No tear	31.9% (119)
	I	15.8% (59)
	II	27.1% (101)
	III	12.6% (47)
	IV	8.8% (33)
	V	3.8% (14)

BMI, body mass index; DM; diabetes mellitus; HL; hyperlipidemia; SSP–ISP: supraspinatus–infraspinatus tendon; SSc: subscapularis tendon.

Table 2 shows the combined status of SSP–ISP tears and SSc tears. The most common combinations were SSP–ISP medium tear only or SSP–ISP medium/large tears with Lafosse Type II SSc tears (Table 2).

**Table 2:** Coexistence of SSP–ISP and SSc tears.

SSc tear severity (Lafosse classification)	SSP–ISP tear size (Modified Cofield classification)					
	No tear	Partial	Small	Medium	Large	Massive
No tear	0	33	6	59	17	4
I	0	11	2	31	11	4
II	1	17	0	42	35	6
III	2	2	1	9	23	10
IV	1	2	1	11	15	3
V	1	2	0	6	4	1

SSP–ISP, supraspinatus–infraspinatus tendon; SSc, subscapularis tendon.

The average time from MRI to surgery was 2.2 months. Regarding the FI grades by the Goutallier classification, Stage 1 was the most common in SSP with 183 cases and 218 cases in ISP, and Grade 0 was the most common in SSc with 227 cases (Table 3). The Cohen *κ* statistics for intraobserver and interobserver reliabilities of the Goutallier classification were 0.81 and 0.71, respectively.

**Table 3:** Proportions of FI grades (Goutallier classification) in rotator cuff muscles.

FI grade (Goutallier classification)	Grade, % (*n*)				
	0	1	2	3	4
Supraspinatus muscle	13.9 (52)	49.1 (183)	27.3 (102)	8.1 (30)	1.6 (6)
Infraspinatus muscle	18.8 (70)	58.4 (218)	15.3 (57)	7.0 (26)	0.5 (2)
Subscapularis muscle	60.9 (227)	22.8 (85)	11.3 (42)	4.0 (15)	1.0 (4)

FI, fatty infiltration.

The associations of the sizes of SSc and SSP–ISP tears with the degree of FI in each rotator cuff muscle were investigated. SSP–ISP tear size was significantly associated not only with the FI grades of the SSP and ISP but also with that of the SSc (Table 4) (*P* < 0.01). SSc tear severity was also significantly associated not only with the FI grade of the SSc but also with those of the SSP and ISP (Table 5) (*P* < 0.01).

**Table 4:** Associations between SSP–ISP tear severity (modified Cofield classification) and fatty infiltration of each rotator cuff muscle.

SSP–ISP tear size (Modified Cofield classification)	Goutallier FI grade, *n*					*P*-value
	0	1	2	3	4	
	Supraspinatus muscle					*P *< 0.01
No tear	3	1	1	0	0	
Partial	21	41	5	0	0	
Small	5	5	0	0	0	
Medium	23	102	31	2	0	
Large	0	31	54	16	4	
Massive	0	3	11	12	2	
	Infraspinatus muscle					*P *< 0.01
No tear	3	2	0	0	0	
Partial	28	39	0	0	0	
Small	5	5	0	0	0	
Medium	29	120	9	0	0	
Large	5	49	38	13	0	
Massive	0	3	10	13	2	
	Subscapularis muscle					*P *< 0.01
No tear	1	1	2	1	0	
Partial	52	9	3	2	1	
Small	9	0	1	0	0	
Medium	108	27	15	7	1	
Large	47	36	16	5	1	
Massive	10	12	5	0	1	

SSP–ISP, supraspinatus–infraspinatus tendon; FI, fatty infiltration.

Results are significant based on the linear-by-linear association test.

**Table 5:** Associations between SSc tear severity (Lafosse classification) and fatty infiltration of the rotator cuff muscles.

SSc tear severity (Lafosse classification)	Goutallier FI grade, *n*					*P*-value
	0	1	2	3	4	
	Supraspinatus muscle					*P *< 0.01
No tear	109	9	1	0	0	
I	49	9	1	0	0	
II	54	36	8	3	0	
III	14	21	10	2	0	
IV	1	10	22	0	0	
V	0	0	0	10	4	
	Infraspinatus muscle					*P *< 0.01
No tear	25	67	20	5	2	
I	13	32	12	2	0	
II	8	48	36	9	0	
III	3	17	16	8	3	
IV	1	14	14	3	1	
V	2	5	4	3	0	
	Subscapularis muscle					*P *< 0.01
No tear	35	66	11	6	1	
I	13	39	4	3	0	
II	14	63	17	6	1	
III	4	20	14	9	0	
IV	3	21	7	2	0	
V	1	9	4	0	0	

SSc, subscapularis tendon; FI, fatty infiltration.

Results are significant based on the linear-by-linear association test.

The associations between the FI grade according to the Goutallier classification and the different patient factors were investigated. Patient age at surgery was significantly associated with the FI grades of the SSP, ISP, and SSc, with the FI grade progressing significantly with advancing age (Table 6). However, there were no significant associations of the FI grade with sex, BMI, presence of DM or HL, and duration of symptoms (Table 7).

**Table 6:** Associations between age and fatty infiltration of each rotator cuff muscle.

Age	Goutallier FI grade, *n*					*P*-value
	0	1	2	3	4	
	Supraspinatus muscle					<0.01
30–39	4	1	1	0	0	
40–49	6	13	2	1	0	
50–59	16	42	20	4	1	
60–69	21	83	52	15	3	
70–79	5	43	26	10	2	
80–	0	1	1	0	0	
	Infraspinatus muscle					<0.01
30–39	4	2	0	0	0	
40–49	11	8	2	1	0	
50–59	20	45	12	5	1	
60–69	27	110	23	13	1	
70–79	8	52	19	7	0	
80–	0	1	1	0	0	
	Subscapularis muscle					<0.01
30–39	6	0	0	0	0	
40–49	20	2	0	0	0	
50–59	58	14	9	2	0	
60–69	103	35	23	9	4	
70–79	40	32	10	4	0	
80–	0	2	0	0	0	

FI, fatty infiltration.

Results are significant based on Spearman's rank correlation coefficient.

**Table 7:** Associations between fatty infiltration of each rotator cuff muscle and sex, BMI, patient comorbidities, and duration of symptoms.

		Goutallier FI grade, *n*					*P*-value
		0	1	2	3	4	
Sex		Supraspinatus muscle					0.41
	Male	38	131	72	21	2	
	Female	14	52	30	9	4	
		Infraspinatus muscle					0.28
	Male	54	151	42	15	2	
	Female	16	67	15	11	0	
		Subscapularis muscle					0.70
	Male	164	54	32	11	3	
	Female	63	31	10	4	1	
BMI (kg/m^2^)		Supraspinatus muscle					0.16
	−20.0	2	18	14	2	0	
	20.1–25.0	30	105	59	18	5	
	25.1–30.0	18	53	25	9	1	
	30.1–	2	7	4	1	0	
		Infraspinatus muscle					0.39
	−20.0	5	24	7	0	0	
	20.1–25.0	38	127	32	19	1	
	25.1–30.0	22	62	15	6	1	
	30.1–	5	5	3	1	0	
		Subscapularis muscle					0.67
	−20.0	21	12	2	1	0	
	20.1–25.0	134	49	24	8	2	
	25.1–30.0	64	22	13	5	2	
	30.1–	8	2	2	1	0	
DM		Supraspinatus muscle					0.33
	DM	7	26	8	5	0	
	Non-DM	45	157	94	25	6	
		Infraspinatus muscle					0.89
	DM	8	28	9	1	0	
	Non-DM	62	190	48	25	2	
		Subscapularis muscle					0.66
	DM	26	13	5	2	0	
	Non-DM	201	72	37	13	4	
HL		Supraspinatus muscle					0.87
	HL	7	22	15	3	1	
	Non-HL	45	161	87	27	5	
		Infraspinatus muscle					0.88
	HL	8	30	9	1	0	
	Non-HL	62	188	48	25	2	
		Subscapularis muscle					0.38
	HL	32	9	6	1	0	
	Non-HL	195	76	36	14	4	
Symptom duration, Mo		Supraspinatus muscle					0.41
	−6	35	115	66	18	4	
	7–12	11	55	19	6	2	
	13–24	6	11	10	2	0	
	25–	0	2	7	4	0	
		Infraspinatus muscle					0.57
	−6	43	142	38	14	1	
	7–12	23	51	10	9	0	
	13–24	4	20	3	2	0	
	25–	0	5	6	1	1	
		Subscapularis muscle					0.40
	−6	140	55	31	9	3	
	7–12	64	17	5	6	1	
	13–24	18	8	3	0	0	
	25–	5	5	3	0	0	

BMI, body mass index; DM, diabetes mellitus; HL, hyperlipidemia; FI, fatty infiltration.

Results are not significant based on Mann–Whitney's *U*-test and Spearman's rank correlation coefficient.

There was no significant difference in FI of each rotator cuff muscle between traumatic and non-traumatic cases. In addition, there was no significant correlation between the time from injury to MRI and FI of the muscle in traumatic cases (Table 8).

**Table 8:** Comparison between traumatic and non-traumatic cases with the Goutallier classification, and associations between fatty infiltration of each rotator cuff muscle and trauma–MRI duration (traumatic cases).

		Goutallier FI grade, *n*					*P*-value
		0	1	2	3	4	
Trauma history		Suprasupinatus muscle					0.90
	Traumatic	20	73	38	14	2	
	Non-traumatic	32	110	64	16	4	
		Infraspinatus muscle					0.93
	Traumatic	29	83	26	8	1	
	Non-traumatic	16	67	15	11	0	
		Subscapularis muscle					0.82
	Traumatic	89	32	18	7	1	
	Non-traumatic	138	53	24	8	3	
Trauma–MRI duration, months (traumatic cases)		Suprasupinatus muscle					0.9
	−2	14	53	27	11	1	
	3–6	4	16	7	2	1	
	6–12	1	4	4	1	0	
	12–	1	0	0	0	0	
		Infraspinatus muscle					0.25
	−2	17	63	20	5	1	
	3–6	9	15	4	2	0	
	6–12	3	4	2	1	0	
	12–	0	1	0	0	0	
		Subscapularis muscle					0.31
	−2	61	26	13	5	1	
	3–6	20	5	4	1	0	
	6–12	7	1	1	1	0	
	12–	1	0	0	0	0	

Results are not significant based on Mann–Whitney's *U*-test and Spearman's rank correlation coefficient.

## Discussion

In the present study, the associations between FI grades and SSc or SSP–ISP tear size, as well as the various patient factors, were investigated. It was found that there were significant associations between SSc tear size and the FI grade of the SSc itself and between SSP–ISP tear size and the FI grade of the SSP–ISP itself. Furthermore, it was shown that SSc tear size was associated with the FI grades of the SSP and the ISP and that SSP–ISP tear size was associated with the FI grade of the SSc. There were some patients in the present study in whom the FI grade of the ISP was comparatively mild, but the FI grades of the SSP and the SSc were more advanced (Figure 1A), and others in whom the FI grade of the ISP was advanced, but the FI grades of the SSP and the SSc were comparatively mild (Figure 2A). Based on the results of the present study, the severity of adjacent rotator cuff tears including the SSc may affect the FI grade of the SSP and ISP in these cases. In clinical settings, we should consider the effect of any tear in the adjacent rotator cuff when assessing the FI grade of the rotator cuff muscles.

The reason for the associations between the size of the tear in each rotator cuff and the FI of the adjacent rotator cuff found in this study is thought to be its anatomical structure. Because the attachments of the SSc and the SSP are close to each other, their disruption may affect the FI of each of these muscles. In recent years, the anatomical structure of the anterior–superior part of the rotator cuff has been clarified, and many studies have testified to its clinical importance. Mochizuki et al. (10) found that the insertion area of the SSP is located at the anteromedial region of the highest impression on the greater tuberosity and is sometimes located at the superior-most area on the lesser tuberosity. Arai et al. (9) conducted a detailed investigation of the insertion of the tendinous portion of the SSc into the lesser tuberosity and reported that, in most cases, the superior-most insertion was located on the superior margin of the lesser tuberosity and that a “thin tendinous slip” extended further upward. These results indicate a close relationship between the SSc and the SSP attachment around the bicipital groove (upper side of the lesser tuberosity and anterior portion of the greater tuberosity). In terms of clinical studies, a prospective study of non-traumatic rotator cuff tears conducted by Hebert-Davies et al. (8) reported that FI was three times more likely to be advanced when damage extended into the anterior portion of the SSP than in cases when the anterior part was preserved. The results of these studies are indicative of both the importance of the anterior fibers of the SSP tendon and its close relationship with the superior part of the SSc tendon, and their anterior and superior insertions may play important roles in the proper functioning of these muscles. The present study demonstrated significant associations between the FI grades in the SSP and the ISP with SSc tear size, results that are not inconsistent with the anatomical and clinical findings described above. These results suggest that the FI grades of the SSP and the ISP may be affected not only by the size of tears in these muscles themselves but also by whether there is any collapse in the anterior–superior part, including the lesser tuberosity into which the SSc inserts. It is difficult to explain the relationship between SSc and ISP tear size and FI, which are not adjacent to each other. This may be due to the fact that SSP and ISP form a layered structure and are connected to each other (10).

The limitations of this study include its nature as a retrospective study that only investigated patients who underwent a variety of arthroscopic surgeries and excluded severe FI cases for which reverse shoulder arthroplasty was indicated. For these reasons, it was not possible to examine the relationship between FI and postoperative cuff integrity of the adjacent rotator cuff. Similarly, although this retrospective study showed an indirect association between adjacent rotator cuff tears and rotator cuff muscle FI, prospective longitudinal studies are needed to directly demonstrate their relationship. Another limitation of the statistical analysis is that some cells in the table had a small sample size, making multivariate analysis difficult. In the present study, SSc and SSP–ISP tears were classified and investigated based on preoperative MRI and arthroscopic findings, but in the case of large or massive tears, the size of the tear may have affected the FI of the entire rotator cuff muscles.

## Conclusions

The results of the present study not only confirmed the previously reported association between rotator cuff tear size and FI grade but also identified associations between SSc tear size and the FI grades of the SSP and ISP and between SSP–ISP tear size and the FI grade of the SSc. The FI grade of each rotator cuff muscle depended not only on the size of the tear in the tendon itself but also on whether there was a tear in the adjacent part of the rotator cuff including the SSc. In light of this finding, the effect of any tear in the adjacent rotator cuff must be considered when assessing the FI grade of the rotator cuff muscles in clinical settings.

## Data Availability

The original contributions presented in the study are included in the article/Supplementary Material, further inquiries can be directed to the corresponding author.
